# Development and validation of a clinical prediction model for the success of focused ultrasound ablation system for the treatment of adenomyosis

**DOI:** 10.1038/s41598-026-48587-z

**Published:** 2026-06-02

**Authors:** Limei Cui, Gang Zhang, Changmei Sang, Zhiyan Wu, Ruoqing Li, Shuping Zhao

**Affiliations:** 1 Department of Gynecology, Qingdao Women and Children’s Hospital, Shandong University, Qingdao, 266071 Shandong China; 2 Department of Gynecology, Qingzhou People’s Hospital, WeiFang, 262500 Shandong China; 3 Department of Thoracic Surgery, Qingzhou People’s Hospital, WeiFang, 262500 Shandong China; 4 Medical College of Qingdao University, Qingdao, 266071 Shandong China

**Keywords:** Adenomyosis, Focused Ultrasound Ablation System, Predictive modelling, Estrogen, Therapeutic dose, Diseases, Medical research

## Abstract

This study aimed to develop and validate a clinical prediction model for the success of the focused ultrasound ablation system (FUAS) in treating adenomyosis. A retrospective analysis was conducted on 250 patients from Qingdao Women and Children’s Hospital (2019–2022). Patients were categorized into success (*n* = 108) or failure (*n* = 142) groups based on a post-treatment lesion ablation rate greater than 80%. The dataset was split into training (70%) and validation (30%) sets. The multivariable analysis identified age (OR = 1.10, *P* = 0.014), depth of adenomyosis (OR = 1.03, *P* = 0.036), and uterine body (OR = 0.25, *P* = 0.027) as independent predictors of FUAS efficacy, which were used to build the model. In the training set, the model achieved an AUC of 0.74 for efficacy prediction. The Bootstrap ROC indicates an AUC mean of 0.751 with a standard deviation of 0.036. In conclusion, a model based on age, depth of ademyosis, and uterine body lesions treatment dose accurately predicts FUAS success for adenomyosis. This tool can aid clinical decision-making and promote personalized treatment.

**Supplementary Information:**

The online version contains supplementary material available at 10.1038/s41598-026-48587-z.

## Introduction

Adenomyosis is a prevalent gynecological condition characterized by the ectopic presence of endometrial tissue within the myometrium, leading to severe dysmenorrhea, heavy menstrual bleeding, and chronic pelvic pain^1^. Adenomyosis affects approximately 20–35% of women of reproductive age, but the condition remains underdiagnosed^2,3^. The significant impact of adenomyosis on women’s quality of life, fertility, and mental health^4,5^. Current interventions, including hormonal therapy and conservative surgery, frequently fail to provide long-term relief or are associated with significant side effects and recurrence of symptoms^6^. Consequently, there is a pressing need for innovative treatment approaches that promise more targeted and effective disease management.

A focused ultrasound ablation system (FUAS) represents a significant advancement in the non-invasive treatment of adenomyosis^7^, generating localized heat to destroy the ectopic endometrial tissue embedded within the myometrium without damaging the surrounding uterine tissue^8^. The precision of FUAS allows for targeted treatment, minimizing the risk of complications associated with more invasive surgical methods^9,10^. The outpatient nature of the procedure, combined with its rapid recovery time, positions FUAS as a preferable option for patients seeking less disruptive treatment alternatives. Despite these advantages, the effectiveness of FUAS can vary significantly among individuals, underscoring the need for predictive models to optimize patient selection and enhance outcomes. Previous research on FUAS ablation for adenomyosis has provided valuable insights into its potential benefits^11–14^; however, these studies have also exposed significant gaps in our understanding and application of this technology^15^.

The primary hypothesis is that a well-constructed clinical prediction model can forecast the success of FUAS ablation in treating adenomyosis based on predefined clinical and demographic parameters. This hypothesis is based on the observation that certain patient-specific factors, including the extent of uterine involvement, age, reproductive history, and estrogen levels, may influence the effectiveness of FUAS ablation.

## Methods

### Study design and patients

This retrospective study included adult female patients diagnosed with adenomyosis from the authors’ hospital between 2019 and 2022. The inclusion criteria were (1) female patients aged 18–60 years, (2) diagnosed with adenomyosis from 2019 to 2022 at the authors’ hospital^16,17^, (3) underwent FUAS ablation, and (4) available estrogen levels measured on cycle day 2–4 of the follicular phase. The exclusion criteria were (1) history of partial hysterectomy for adenomyosis or history of FUAS before the study period, (2) presence of specific comorbid conditions, or (3) pregnancy.

The dataset was split into training (70%) and validation (30%) sets for model development and validation.

### Ethical review and patient consent

The study protocol has been reviewed and approved by the institutional review board (IRB) at the author’s Hospital. All patients provided clinical consent to the FUAS procedure. The ethics committee waived the requirement for individual informed consent for research purposes, given the study’s retrospective nature.

### Data collection and definitions

In this study, comprehensive data were collected from the patient charts. The variables included basic demographic details, such as age, body mass index (BMI), hyperprolactinemia, estrogen levels, CA125, and CA199. Anatomical and morphological measurements were meticulously noted, including uterine and lesion volumes, thickness of the rectus abdominis and subcutaneous fat, and the depth of adenomyosis lesions from the sacrococcygeal junction. Detailed treatment parameters, including maximum, minimum, and average power, treatment and irradiation times, treatment intensity, dose, and volume, were also recorded. Reproductive history was detailed through variables such as parity, symptoms of urinary frequency and constipation, presence of anemia, fertility requirements, history of uterine surgery, reproductive tract anomalies, and family history of adenomyosis or endometriosis. Further, uterine position, scores for heavy menstrual bleeding and dysmenorrhea, and a diagnostic classification system were assessed. Uterine and lesion volumes were calculated as V = 0.5233×a×b×c based on the three-axis measurements from ultrasound or magnetic resonance imaging (MRI) images. Lesion position was categorized as (1) uterine floor (lower segment of the uterus near the cervix on the right side, including the uterine floor, posterior wall, anterior and posterior walls of the uterine floor, anterior and posterior walls of the uterine floor, anterior and posterior walls of the uterine floor, anterior and posterior walls of the uterine floor, right side of the uterine floor, uterine floor, posterior wall, and uterine floor), (2) uterine body (posterior wall, posterior wall, anterior wall, right anterior wall, left posterior wall, right posterior wall, left anterior wall, right anterior wall, right anterior wall, right posterior wall, left anterior wall, right anterior wall, right posterior wall, left anterior wall, posterior wall), and (3) diffuse. Uterine adenomyosis ablation ratio = (volume of non-perfused uterine lesion after treatment/volume of uterine adenomyosis lesion before treatment) × 100%. The success rate of FUAS in treating adenomyosis was assessed as an 80% reduction in lesion volume. The selection of the 80% non-perfused volume ratio (NPVR) threshold as the definition of sufficient ablation is grounded in a well-established body of literature rather than a single arbitrary cutoff. Indeed, the 80% NPVR threshold has been progressively validated through an evolving series of studies. Initially, an NPVR *≥* 60% was used as the efficacy endpoint during early FUAS training programs, as achieving this level yielded re-intervention rates comparable to those of myomectomy. Subsequently, as technical proficiency improved, the threshold for operational training standards was raised to 70%. Park et al. then reported that achieving an immediate NPVR of at least 80% during FUAS of uterine fibroids was safe and associated with significantly greater tumor volume shrinkage (43% reduction) compared with cases achieving an NPVR < 80% (20% reduction). On this basis, multiple investigators have adopted NPVR *≥* 80% as the benchmark for sufficient ablation^18–21^. The energy doses were taken from the ultrasound system data. When analyzing the dose as a continuous variable, using an ablation rate > 80% as the gold standard, and running a ROC curve analysis, it was found that 240 kJ corresponds to the maximum Youden index. Therefore, the treatment dose was dichotomized based on 240 kJ. Routine follow-ups were at 24 h and 1, 3, and 6 months after the procedure. Two physicians with associate senior professional titles or above in the imaging department independently performed the measurements. Thirty cases were randomly selected and independently measured by two senior physicians/technicians in a double-blind manner. ICC (continuous variable) or kappa (categorical variable) was calculated. If ICC were > 0.75 or kappa were > 0.60, good consistency could be considered.

### Statistical analysis

This study encompassed a cohort of 250 patients, comprising 108 events (indicative of treatment success) and 142 non-events (representative of treatment failure). The adequacy of sample size for the development of the predictive model utilizing logistic regression was assessed based on the events-per-variable (EPV) principle. In accordance with widely accepted guidelines (EPV ≥ 10), the available number of events (*n* = 108) permitted the inclusion of up to approximately 10 predictors in the multivariable model, thereby indicating that the present sample size was adequate for reliable model estimation. Furthermore, a post hoc power analysis was performed for the logistic regression. Assuming a two-sided alpha level of 0.05, a sample size of 250, and an event rate of 43.2%, the study demonstrated more than 80% statistical power to identify an odds ratio of approximately 1.8 or greater for the key predictors.

For continuous data, the mean method was adopted for the interpolation of missing data. For categorical variables, the mode interpolation method is used for interpolation. Descriptive statistics were applied to baseline variables, summarizing normally distributed variables using means ± SD and non-normally distributed variables with medians and interquartile ranges. Categorical variables were reported as frequencies and percentages, with comparisons made via t-tests, Mann-Whitney U tests, or chi-squared tests as appropriate. For model development and validation, the sample was divided into a training set (70%) and a test set (30%). The split was performed in R 4.4.1 using a seed (12345). Logistic regression was utilized to develop the model on the training set and validate it on the test set, with groups formed based on treatment outcomes to facilitate univariate analysis. Univariate logistic regression identified potential predictors; variables with p-values < 0.2 advanced to multivariable logistic regression to adjust for confounders and identify independent predictors (stepwise method). The variance inflation factor (VIF) was used to assess multicollinearity among continuous variables, leading to the exclusion or combination of variables with strong multicollinearity, i.e., with a VIF > 10. The bidirectional stepwise regression method was used to include variables in the model, and the variables that contributed significantly to the model were included. The model was subsequently revised to incorporate only the non-multicollinear variables. The discriminative ability of the nomogram was evaluated by the area under the receiver operating characteristic curve (AUC) in both the training and validation sets. To assess the model’s stability and correct for optimism due to the limited training sample size, internal validation was performed using 1000 bootstrap resamples on the training set. This process generated the optimism-corrected AUC and its 95% confidence interval, as well as the bootstrap-corrected calibration slope. All analyses were performed using R 4.4.1.

## Results

### Predictive value of clinical parameters

A total of 250 patients with adenomyosis, aged 27–55 years (median age, 42 years), were included in this study, comprising 175 (70%) in the training set and 75 (30%) in the validation set. They were divided into two groups based on FUAS ablation rate: the failure group (ablation rate < 80%, 142 patients, 56.8%) and the success group (ablation rate ≥ 80%, 108 patients, 43.2%).

Univariate analysis revealed that age (*P* = 0.014) and lesion volume (*P* = 0.029) were statistically significantly different between the FUAS efficacy subgroups. Notably, the median age of patients in the success group (41 years) was significantly lower than that of the failure group (43.5 years). The volume of lesions in the success group (88.29 cm^3^) was significantly smaller than in the failure group (107.48 cm^3^). Table 1 summarizes all the baseline characteristics of the 250 study subjects.

**Table 1:** Characteristics of 250 patients with adenomyosis.

Variables	Total (*n* = 250)	Failure group (*n* = 142)	Success group (*n* = 108)	Statistic	*P*
AGE, M (Q₁, Q₃)	42.00 (37.25, 46.00)	41.00 (37.00, 46.00)	43.50 (39.00, 46.25)	Z=−2.46	**0.014**
BMI, M (Q₁, Q₃)	24.03 (21.78, 26.64)	23.62 (21.88, 26.70)	24.24 (21.77, 26.47)	Z=−0.06	0.951
CA125, M (Q₁, Q₃)	75.90 (40.60, 132.53)	77.85 (39.70, 133.73)	74.70 (42.02, 121.10)	Z=−0.39	0.696
CA199, M (Q₁, Q₃)	21.77 (12.21, 33.47)	23.83 (11.96, 35.89)	19.64 (12.60, 32.01)	Z=−0.88	0.380
Uterine volume, M (Q₁, Q₃)	268921.52 (205903.38, 381535.94)	251776.38 (194607.16, 368403.79)	288630.83 (219955.55, 420274.79)	Z=−1.78	0.075
Lesion volume, M (Q₁, Q₃)	94812.02 (67278.06, 148496.84)	88291.18 (60539.01, 141621.86)	107477.45 (75640.92, 160006.17)	Z=−2.19	**0.029**
Rectus abdominis thickness, M (Q₁, Q₃)	9.00 (7.00, 11.00)	9.00 (7.00, 11.00)	9.00 (7.00, 10.00)	Z=−1.16	0.244
Subcutaneous fat thickness, M (Q₁, Q₃)	16.00 (12.00, 21.75)	18.00 (13.00, 22.00)	15.00 (11.00, 21.00)	Z=−1.65	0.099
Depth of adenomyosis lesions, M (Q₁, Q₃)	14.00 (8.00, 24.00)	13.00 (8.00, 23.75)	14.50 (8.00, 24.75)	Z=−0.41	0.684
Average power, M (Q₁, Q₃)	400.00 (398.00, 400.00)	400.00 (394.00, 400.00)	400.00 (399.00, 400.00)	Z=−1.94	0.053
Treat Time, M (Q₁, Q₃)	73.00 (55.00, 97.75)	71.00 (55.25, 100.25)	74.50 (54.75, 96.00)	Z=−0.03	0.977
Irradiation time, M (Q₁, Q₃)	600.00 (401.00, 859.50)	600.00 (416.75, 889.50)	611.50 (387.75, 836.75)	Z=−0.11	0.910
Treat Intensity, M (Q₁, Q₃)	480.00 (421.00, 559.75)	474.50 (411.75, 550.00)	491.50 (425.50, 570.75)	Z=−0.89	0.372
Treat volume, M (Q₁, Q₃)	2.90 (2.30, 3.80)	2.80 (2.20, 3.88)	3.10 (2.30, 3.80)	Z=−0.82	0.410
High estrogen level, n (%)				χ²=0.32	0.570
0	176 (70.40)	102 (71.83)	74 (68.52)		
1	74 (29.60)	40 (28.17)	34 (31.48)		
Treatment dose, n (%)				χ²=0.02	0.885
< 240	124 (49.60)	71 (50.00)	53 (49.07)		
≥ 240	126 (50.40)	71 (50.00)	55 (50.93)		
Maximum power, n (%)				χ²=1.70	0.192
350	12 (4.80)	9 (6.34)	3 (2.78)		
400	238 (95.20)	133 (93.66)	105 (97.22)		
Minimum power, n (%)				-	0.635
300	4 (1.60)	2 (1.41)	2 (1.85)		
350	91 (36.40)	55 (38.73)	36 (33.33)		
400	155 (62.00)	85 (59.86)	70 (64.81)		
Number of births, n (%)				-	0.402
0	35 (14.00)	24 (16.90)	11 (10.19)		
1	140 (56.00)	74 (52.11)	66 (61.11)		
2	68 (27.20)	40 (28.17)	28 (25.93)		
3	6 (2.40)	3 (2.11)	3 (2.78)		
4	1 (0.40)	1 (0.70)	0 (0.00)		
Urinary frequency and constipation, n (%)				-	> 0.999
No	248 (99.20)	141 (99.30)	107 (99.07)		
Yes	2 (0.80)	1 (0.70)	1 (0.93)		
Anemia, n (%)				χ²=2.93	0.087
No	122 (48.80)	76 (53.52)	46 (42.59)		
Yes	128 (51.20)	66 (46.48)	62 (57.41)		
Fertility requirements, n (%)				χ²=1.71	0.191
No	184 (73.60)	100 (70.42)	84 (77.78)		
Yes	66 (26.40)	42 (29.58)	24 (22.22)		
History of uterine surgery, n (%)				χ²=1.09	0.296
No	167 (66.80)	91 (64.08)	76 (70.37)		
Yes	83 (33.20)	51 (35.92)	32 (29.63)		
History of reproductive tract anomalies causing obstruction, n (%)				-	> 0.999
No	248 (99.20)	141 (99.30)	107 (99.07)		
Yes	2 (0.80)	1 (0.70)	1 (0.93)		
Family history of adenomyosis or endometriosis, n (%)				χ²=1.70	0.192
No	238 (95.20)	133 (93.66)	105 (97.22)		
Yes	12 (4.80)	9 (6.34)	3 (2.78)		
Hyperprolactinemia, n (%)				χ²=0.60	0.440
No	230 (92.00)	129 (90.85)	101 (93.52)		
Yes	20 (8.00)	13 (9.15)	7 (6.48)		
Uterine position, n (%)				-	0.248
Anterior	193 (77.20)	105 (73.94)	88 (81.48)		
Mid-position	56 (22.40)	36 (25.35)	20 (18.52)		
Posterior	1 (0.40)	1 (0.70)	0 (0.00)		
Lesion position, n (%)				-	0.485
Uterine floor	30 (12.00)	14 (9.86)	16 (14.81)		
Uterine body	218 (87.20)	127 (89.44)	91 (84.26)		
Diffuse	2 (0.80)	1 (0.70)	1 (0.93)		
Pregnancy number, n (%)				-	0.216*
0	14 (5.60)	10 (7.04)	4 (3.70)		
1	41 (16.40)	27 (19.01)	14 (12.96)		
2	47 (18.80)	24 (16.90)	23 (21.30)		
3	62 (24.80)	32 (22.54)	30 (27.78)		
4	46 (18.40)	26 (18.31)	20 (18.52)		
5	23 (9.20)	10 (7.04)	13 (12.04)		
6	8 (3.20)	7 (4.93)	1 (0.93)		
7	6 (2.40)	4 (2.82)	2 (1.85)		
8	2 (0.80)	2 (1.41)	0 (0.00)		
9	1 (0.40)	0 (0.00)	1 (0.93)		
Heavy menstrual bleeding score, n (%)				-	0.929
0	35 (14.00)	20 (14.08)	15 (13.89)		
1	5 (2.00)	4 (2.82)	1 (0.93)		
2	46 (18.40)	28 (19.72)	18 (16.67)		
3	64 (25.60)	34 (23.94)	30 (27.78)		
4	52 (20.80)	28 (19.72)	24 (22.22)		
5	46 (18.40)	27 (19.01)	19 (17.59)		
6	2 (0.80)	1 (0.70)	1 (0.93)		
Dysmenorrhea score, n (%)				χ²=10.71	0.296
0	12 (4.80)	5 (3.52)	7 (6.48)		
1	20 (8.00)	12 (8.45)	8 (7.41)		
2	1 (0.40)	0 (0.00)	1 (0.93)		
3	20 (8.00)	13 (9.15)	7 (6.48)		
4	17 (6.80)	5 (3.52)	12 (11.11)		
5	22 (8.80)	15 (10.56)	7 (6.48)		
6	46 (18.40)	27 (19.01)	19 (17.59)		
7	24 (9.60)	15 (10.56)	9 (8.33)		
8	65 (26.00)	35 (24.65)	30 (27.78)		
9	23 (9.20)	15 (10.56)	8 (7.41)		
Type, n (%)				-	0.759
1	64 (25.60)	33 (23.24)	31 (28.70)		
2	44 (17.60)	28 (19.72)	16 (14.81)		
3	9 (3.60)	4 (2.82)	5 (4.63)		
4	109 (43.60)	62 (43.66)	47 (43.52)		
5	4 (1.60)	3 (2.11)	1 (0.93)		
6	20 (8.00)	12 (8.45)	8 (7.41)		

Z: Mann-Whitney test, χ²: Chi-square test, -: Fisher exact, *: Simulated p-value. M: Median, Q₁: 1 st Quartile, Q₃: 3 st Quartile. Significant values (P<0.05) are in bold.

### Establishment and validation of clinical models

Variables that were statistically significant in the above univariate analyses were further included in both univariate and multivariable logistic regression analyses to predict the success of FUAS in treating adenomyosis. These variables included age, lesion volume, estrogen level, mean power, and treatment dose.

In the univariate logistic regression analyses, age (OR = 1.08, *P* = 0.004), one birth (OR = 3.01, *P* = 0.045), and uterine body (OR = 0.33, *P* = 0.032) were predictive of FUAS efficacy in patients with adenomyosis (Table 2). All VIFs were < 10, meaning that no variables were excluded on the basis of multicollinearity (Supplementary Table S1). In the multivariable logistic regression analysis, age (OR = 1.10, *P* = 0.014), depth of adenomyosis lesions (OR = 1.03, *P* = 0.036), and uterine body lesions (OR = 0.25, *P* = 0.027) demonstrated independent predictive value for FUAS efficacy in adenomyosis (Table 2).

**Table 2:** Univariate and multivariable Logistic regression analysis for predicting the efficacy of FUAS in adenomyosis patients in the training set.

Variables	Univariate					Multivariable				
	β	S.E	Z	*P*	OR (95%CI)	β	S.E	Z	*P*	OR (95%CI)
AGE	0.08	0.03	2.89	**0.004**	1.08 (1.03 ~ 1.14)	0.10	0.04	2.47	**0.014**	1.10 (1.02 ~ 1.19)
BMI	0.02	0.04	0.42	0.674	1.02 (0.93 ~ 1.11)					
CA125	−0.00	0.00	−0.64	0.519	1.00 (1.00 ~ 1.00)					
CA199	−0.00	0.01	−0.82	0.412	1.00 (0.99 ~ 1.01)					
Uterine volume	−0.00	0.00	−0.43	0.666	1.00 (1.00 ~ 1.00)					
Lesion volume	0.00	0.00	0.56	0.575	1.00 (1.00 ~ 1.00)					
Rectus abdominis thickness	0.01	0.05	0.12	0.903	1.01 (0.91 ~ 1.12)					
Subcutaneous fat thickness	−0.02	0.02	−0.78	0.433	0.98 (0.95 ~ 1.02)					
Depth of adenomyosis lesions	0.02	0.01	1.34	0.180	1.02 (0.99 ~ 1.04)	0.03	0.01	2.09	**0.036**	1.03 (1.01 ~ 1.06)
Maximum power										
350					1.00 (Reference)					1.00 (Reference)
400	1.79	1.08	1.66	0.098	5.99 (0.72 ~ 49.76)	2.19	1.36	1.61	0.107	8.95 (0.62 ~ 128.96)
Minimum power										
300					1.00 (Reference)					
350	−0.33	1.03	−0.32	0.753	0.72 (0.10 ~ 5.47)					
400	−0.17	1.02	−0.16	0.871	0.85 (0.12 ~ 6.24)					
Average power	0.02	0.01	1.85	0.065	1.02 (1.00 ~ 1.05)	0.00	0.02	0.04	0.968	1.00 (0.97 ~ 1.03)
Treat Time	0.00	0.00	0.81	0.416	1.00 (1.00 ~ 1.01)					
Irradiation time	0.00	0.00	0.29	0.769	1.00 (1.00 ~ 1.00)					
Treat Intensity	0.00	0.00	0.63	0.527	1.00 (1.00 ~ 1.00)					
Treat volume	0.03	0.12	0.29	0.774	1.04 (0.82 ~ 1.32)					
Treatment Dose										
< 240					1.00 (Reference)					
≥ 240	0.36	0.31	1.18	0.238	1.43 (0.79 ~ 2.61)					
Number of births										
0					1.00 (Reference)					1.00 (Reference)
1	1.10	0.55	2.00	**0.045**	3.01 (1.02 ~ 8.86)	1.35	0.71	1.91	0.056	3.86 (0.97 ~ 15.41)
2	1.00	0.59	1.71	0.087	2.73 (0.86 ~ 8.59)	1.38	0.81	1.70	0.090	3.96 (0.81 ~ 19.41)
3	1.16	1.12	1.04	0.301	3.20 (0.35 ~ 28.94)	0.91	1.35	0.67	0.501	2.48 (0.18 ~ 34.73)
4	−13.40	882.74	−0.02	0.988	0.00 (0.00 ~ Inf)	−15.73	1455.40	−0.01	0.991	0.00 (0.00 ~ Inf)
Urinary frequency and constipation										
No					1.00 (Reference)					
Yes	14.80	882.74	0.02	0.987	2668356.59 (0.00 ~ Inf)					
Anemia										
No					1.00 (Reference)					1.00 (Reference)
Yes	0.49	0.31	1.59	0.112	1.63 (0.89 ~ 2.99)	0.46	0.37	1.26	0.207	1.59 (0.77 ~ 3.24)
Fertility requirements										
No					1.00 (Reference)					1.00 (Reference)
Yes	−0.48	0.37	−1.32	0.187	0.62 (0.30 ~ 1.26)	1.13	0.60	1.89	0.058	3.10 (0.96 ~ 10.01)
History of uterine surgery										
No					1.00 (Reference)					
Yes	−0.19	0.32	−0.60	0.550	0.82 (0.44 ~ 1.56)					
History of reproductive tract anomalies causing obstruction										
No					1.00 (Reference)					
Yes	14.80	882.74	0.02	0.987	2668356.59 (0.00 ~ Inf)					
Family history of adenomyosis or endometriosis										
No					1.00 (Reference)					
Yes	−0.49	0.88	−0.56	0.577	0.61 (0.11 ~ 3.43)					
Hyperprolactinemia										
No					1.00 (Reference)					
Yes	0.09	0.54	0.17	0.864	1.10 (0.38 ~ 3.17)					
High estrogen level										
No					1.00 (Reference)					
Yes	0.03	0.33	0.09	0.928	1.03 (0.54 ~ 1.98)					
Uterine position										
Anterior					1.00 (Reference)					1.00 (Reference)
Mid-position	−0.60	0.36	−1.65	0.100	0.55 (0.27 ~ 1.12)	−0.18	0.44	−0.42	0.675	0.83 (0.35 ~ 1.96)
Posterior	−14.50	882.74	−0.02	0.987	0.00 (0.00 ~ Inf)	−15.12	1455.40	−0.01	0.992	0.00 (0.00 ~ Inf)
Lesion position										
Uterine floor					1.00 (Reference)					1.00 (Reference)
Uterine body	−1.11	0.52	−2.14	**0.032**	0.33 (0.12 ~ 0.91)	−1.39	0.63	−2.21	**0.027**	0.25 (0.07 ~ 0.85)
Diffuse	−0.77	1.50	−0.52	0.606	0.46 (0.02 ~ 8.69)	−1.84	1.58	−1.16	0.247	0.16 (0.01 ~ 3.56)
Pregnancy number										
0					1.00 (Reference)					
1	−0.59	0.87	−0.68	0.499	0.56 (0.10 ~ 3.05)					
2	0.29	0.80	0.36	0.720	1.33 (0.28 ~ 6.44)					
3	0.69	0.79	0.88	0.381	2.00 (0.42 ~ 9.42)					
4	0.45	0.80	0.56	0.573	1.57 (0.33 ~ 7.62)					
5	0.76	0.89	0.86	0.390	2.14 (0.38 ~ 12.20)					
6	−1.10	1.32	−0.83	0.404	0.33 (0.03 ~ 4.40)					
7	0.11	1.17	0.09	0.928	1.11 (0.11 ~ 10.99)					
8	−14.06	882.74	−0.02	0.987	0.00 (0.00 ~ Inf)					
Heavy menstrual bleeding score										
0					1.00 (Reference)					
1	−16.06	1199.77	−0.01	0.989	0.00 (0.00 ~ Inf)					
2	0.13	0.55	0.24	0.813	1.14 (0.38 ~ 3.38)					
3	0.55	0.51	1.08	0.280	1.74 (0.64 ~ 4.75)					
4	0.51	0.54	0.94	0.347	1.67 (0.57 ~ 4.84)					
5	0.33	0.55	0.60	0.549	1.39 (0.47 ~ 4.06)					
6	−16.06	2399.54	−0.01	0.995	0.00 (0.00 ~ Inf)					
Dysmenorrhea score	−0.09	0.06	−1.44	0.150	0.92 (0.81 ~ 1.03)	−0.05	0.07	−0.73	0.463	0.95 (0.83 ~ 1.09)
Classification										
1					1.00 (Reference)					1.00 (Reference)
2	−0.63	0.50	−1.26	0.206	0.53 (0.20 ~ 1.41)	−0.22	0.58	−0.38	0.706	0.80 (0.26 ~ 2.50)
3	−0.19	0.77	−0.25	0.805	0.83 (0.18 ~ 3.75)	−0.14	0.96	−0.14	0.886	0.87 (0.13 ~ 5.69)
4	−0.46	0.39	−1.18	0.239	0.63 (0.30 ~ 1.35)	−0.59	0.44	−1.34	0.180	0.56 (0.24 ~ 1.31)
5	−1.29	1.20	−1.08	0.281	0.28 (0.03 ~ 2.87)	−1.81	1.32	−1.37	0.169	0.16 (0.01 ~ 2.17)
6	−0.80	0.59	−1.34	0.180	0.45 (0.14 ~ 1.45)	−1.20	0.67	−1.81	0.070	0.30 (0.08 ~ 1.10)

OR: Odds Ratio, CI: Confidence Interval. Significant values (P<0.05) are in bold.

**Fig. 1:**
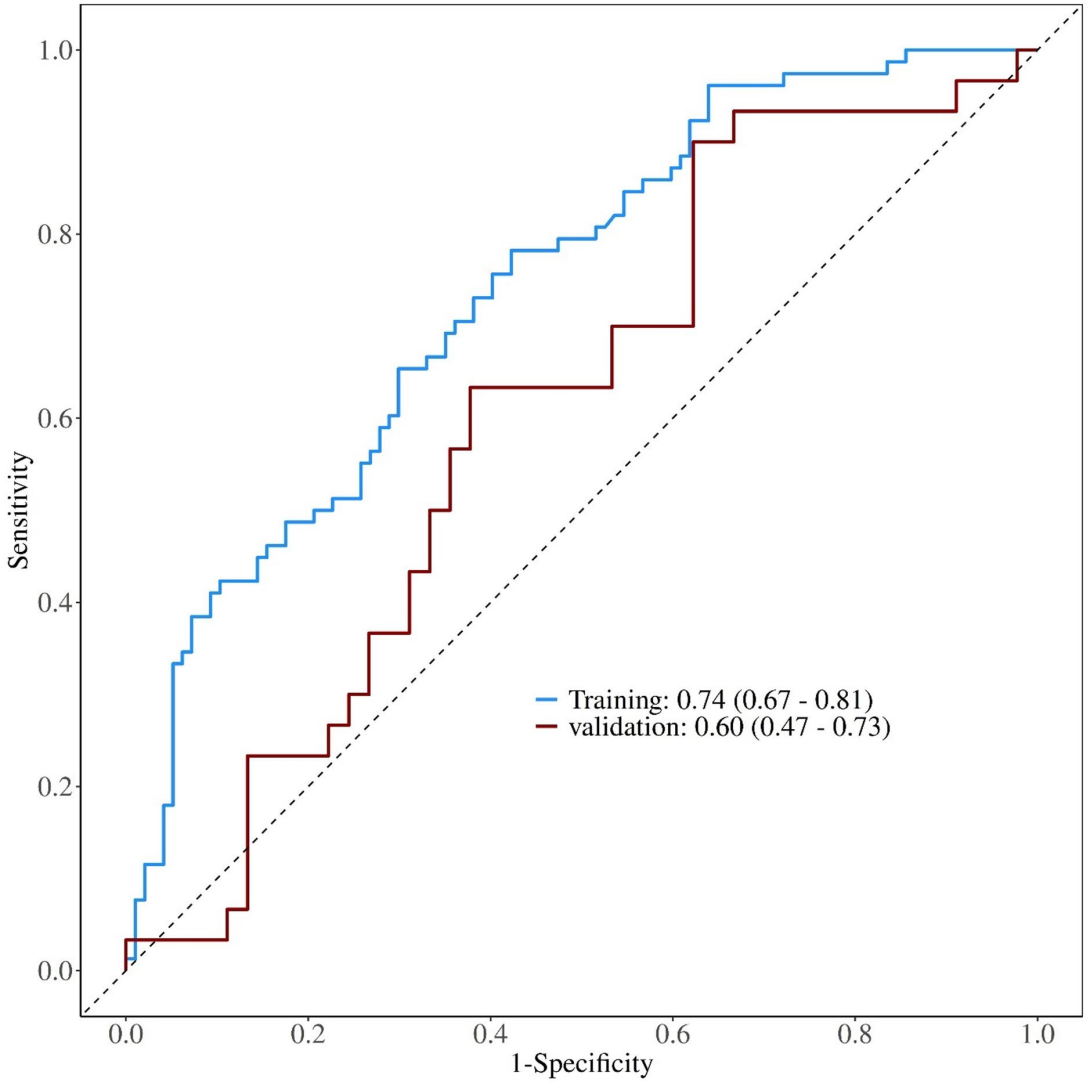
ROC for training set and validation set.

**Fig. 2:**
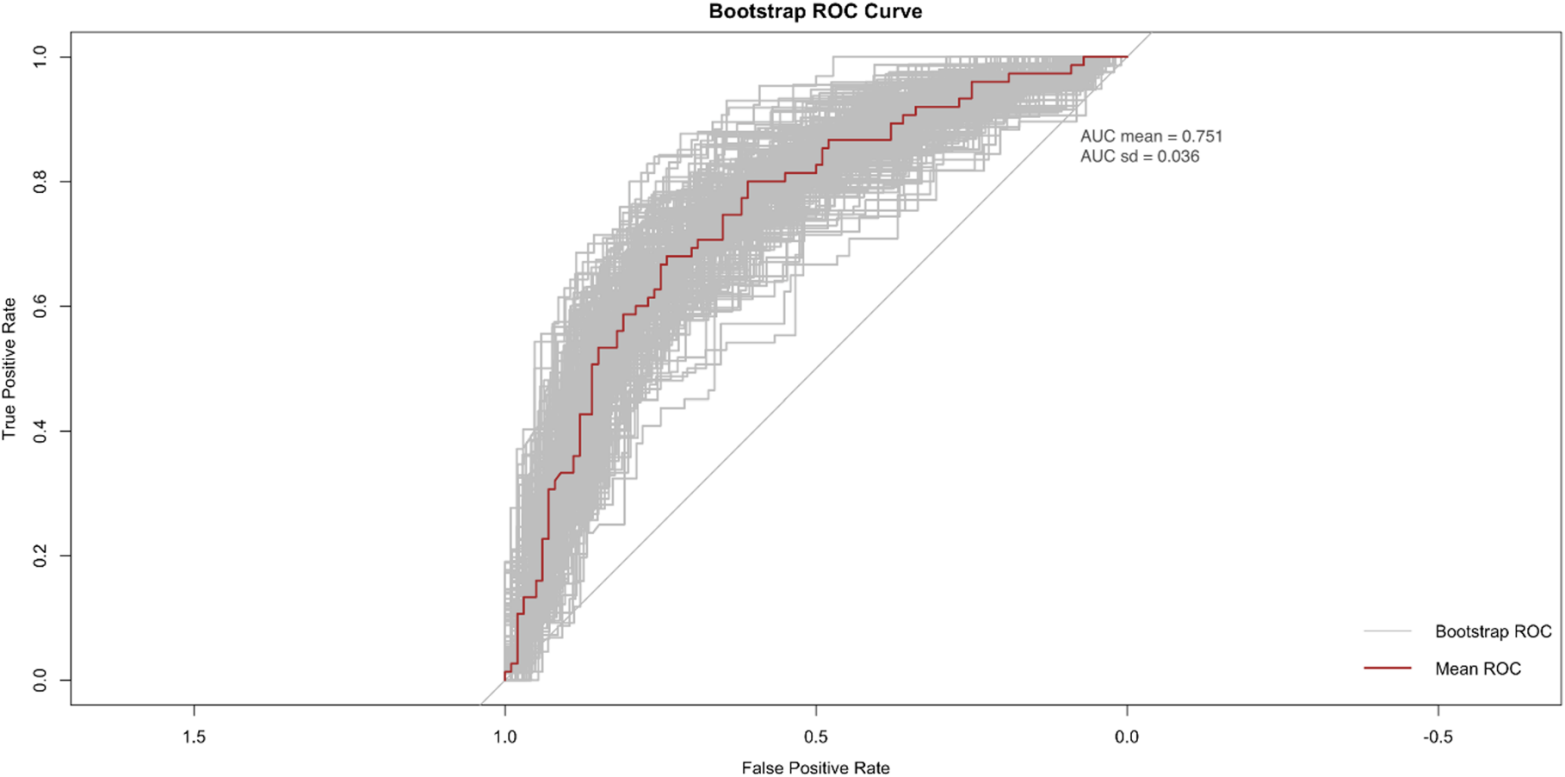
ROC for bootstrap in validation set.

**Fig. 3:**
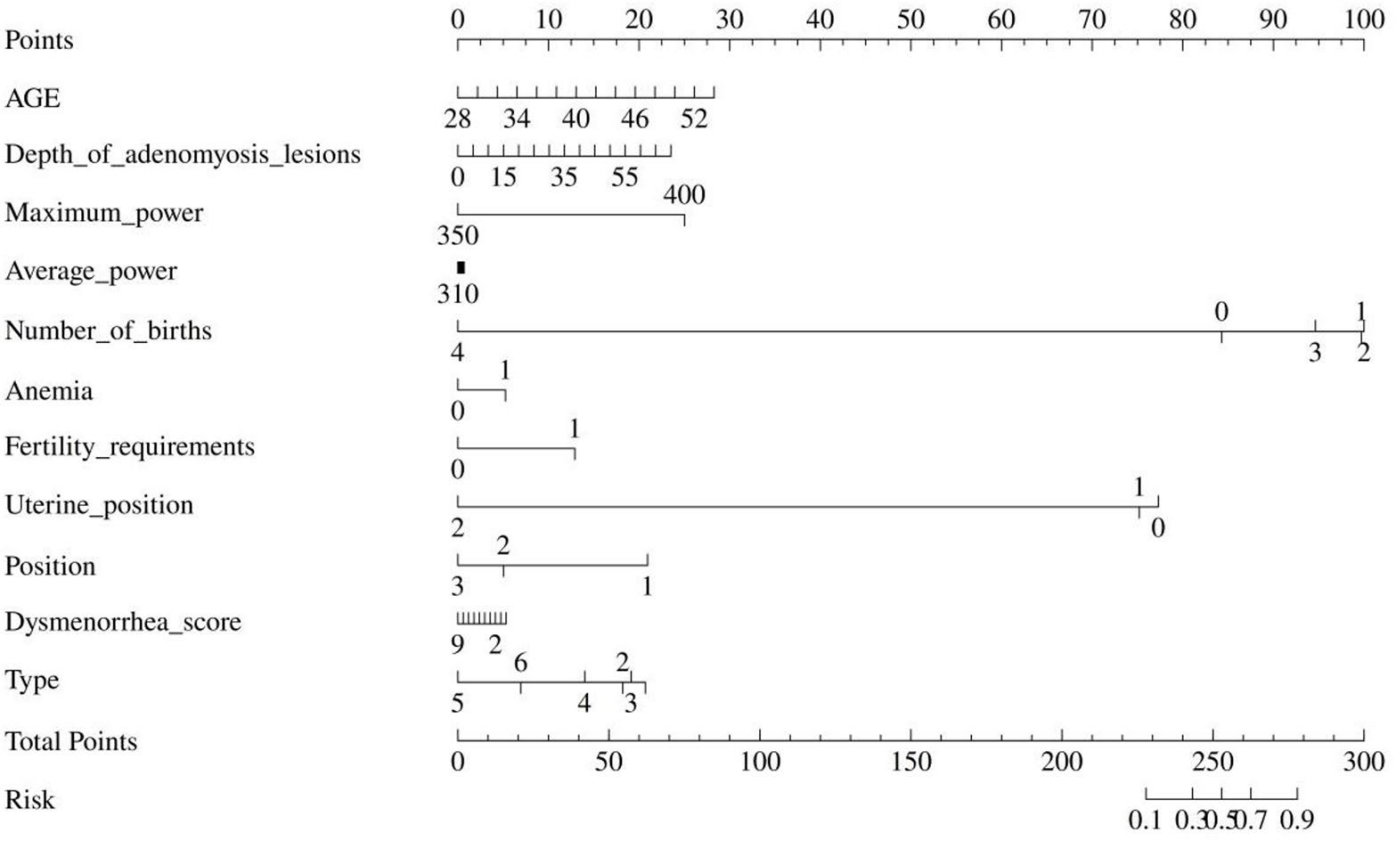
Nomogram. (1) Locate each predictor on its corresponding axis and mark the patient’s value. (2) For each predictor, draw a vertical line up to the “Points” axis to obtain the individual point score. (3) Sum the points from all predictors to obtain the “Total points.” (4) Find the “Total points” value on the total-points axis and draw a vertical line down to the outcome (e.g., risk or survival probability) scale. (5) Read off the predicted probability and interpret it in the context of clinical judgment and other relevant information.

A nomogram was constructed based on the independent predictors in the multivariable logistic regression analysis. The nomogram model predicted FUAS efficacy in patients with adenomyosis, with AUCs of 0.74 in the training set and 0.60 in the validation set (Fig. 1). The “Bootstrap ROC Curve” indicates an AUC mean of 0.751 with a standard deviation of 0.036, demonstrating the model’s predictive performance and its variability across bootstrap samples (Fig. 2). The nomogram is shown in Fig. 3, and the intercept values are shown in Supplementary Table S2. The model shows generally good bootstrap calibration in the training set, with predicted probabilities close to observed outcomes overall, but a tendency to slightly underestimate risk at low predicted probabilities and overestimate risk at high predicted probabilities. In the validation set, the model is fairly well calibrated overall but tends to underestimate risk at lower predicted probabilities and increasingly overestimate risk at higher predicted probabilities (Supplementary Figure S1).

## Discussion

Adenomyosis is a common clinical gynecological disease with an overall prevalence of approximately 0.79% worldwide^22^. Despite its high prevalence and its correlation with the pathogenesis of endometriosis, the pathogenesis and pathophysiological processes of adenomyosis remain poorly understood, which, to some extent, hinders the development of more effective treatment options^23^. Currently, treatments for adenomyosis include pharmacological treatments (painkillers, hormonal drugs), interventional treatments (uterine artery embolization), and surgical treatments (lesion excision, hysterectomy). However, these methods have limitations in terms of efficacy and side effects. Pharmacological treatments typically only alleviate symptoms, making it challenging to cure the disease at its root^24^. Surgical treatments, although effective in removing the lesions, may have an impact on fertility, especially for women with reproductive needs. Therefore, ensuring treatment efficacy while preserving patient fertility is a major challenge in the management of adenomyosis.

The emergence of FUAS technology has brought new hope for the treatment of adenomyosis. FUAS utilizes the focusing properties of ultrasound to accurately concentrate energy at the lesion site, causing coagulative necrosis of the diseased tissue through thermal effects, thereby achieving therapeutic goals^25^. Its main advantages and features include: non-invasiveness, targeted delivery, significant efficacy, reproducibility, and individualized treatment. For patients with adenomyosis who do not wish to undergo surgery or who are not suitable for surgery, FUAS is an ideal alternative treatment option, especially in preserving fertility. FUAS treatment can significantly improve the symptoms of patients with adenomyosis and have a positive impact on their fertility^13^. Compared with drug therapy alone, FUAS combined with drug therapy can solve the problem at the root, reduce the possibility of recurrence to a greater extent, and provide patients with more durable efficacy. Dai et al.^26^ explored the value of FUAS in combination with various drugs for the treatment of adenomyosis and found that FUAS, combined with dienogest or GnRH-a, was effective in reducing pain and anemia and had a low risk of recurrence. The development of FUAS technology has also contributed to improvements in symptoms in patients with adenomyosis. The development of FUAS technology also advances precision medicine for adenomyosis, enabling patients to receive more personalized treatment plans through more accurate diagnosis and treatment.

Adenomyosis is characterized by significant heterogeneity among individuals^27^. Yildiz et al.^28^ found a high degree of heterogeneity of fibroblast-like cells in adenomyosis. Therefore, the clinical presentation, disease severity, and response to treatment may vary significantly among patients with adenomyosis. The traditional ‘one-size-fits-all’ treatment model often fails to meet the diverse needs of all patients. In this study, we leveraged clinical information to develop an individualized risk assessment and efficacy prediction model that guides clinicians in selecting the most suitable treatment options. We constructed a prediction model that included three parameters (age, uterine body lesions, and depth of adenomyosis) based on the results of the multivariable logistic regression analysis. The model predicted FUAS efficacy in patients with adenomyosis, with AUCs of 0.74 and 0.60 in the training and validation sets, respectively. A combined non‑contrast MRI radiomics + clinical model in 130 adenomyosis patients predicted high vs. low ablation rate (NPVR *≥* 50% vs. < 50%); the radiomics and combined models outperformed a purely clinical-imaging model and showed superior net benefit on DCA^29^. A 2025 study developed a joint T2WI‑FS radiomics + clinical model (decision tree/random forest) to predict energy efficiency factor (EEF) requirements for FUAS treatment of adenomyosis, enabling preoperative estimation of energy dose and procedural difficulty, with good performance in both training and test cohorts^30^.

Age influences both clinical success and the durability of symptom relief after FUAS for adenomyosis, but its impact depends on the outcome of interest (symptom control vs. fertility) and interacts with treatment parameters, such as the non‑perfused volume ratio. There is evidence that older age is associated with better clinical outcomes, supporting the present study. In a retrospective cohort of 230 women treated with ultrasound‑guided FUAS, age *≥* 40 years was independently associated with higher long‑term clinical success (89.0% vs. 78.6% in those < 40 years; OR for younger age: 0.342, 95% CI 0.143–0.819)^31^. The same study reported that older age and a higher non‑perfused volume (NPV) ratio (extent of ablation) were the main predictors of durable symptom relief; higher BMI and lower acoustic power increased the risk of recurrence. The authors hypothesized that younger women have higher estrogen levels and more aggressive lesion biology, and often receive more conservative ablation to preserve fertility, resulting in lower NPV ratios and reduced clinical durability^31^. In practice, this means that middle‑aged, perimenopausal women in whom a more extensive ablation is acceptable may experience more sustained symptom control after focused ultrasound. A 2024 review of high‑intensity focused ultrasound for adenomyosis reported that younger age, lower BMI, internal (vs. external) adenomyosis, higher non‑perfused volume ratio, and shorter infertility duration were associated with better reproductive outcomes following FUAS^12^. The review explicitly suggests treating at an earlier age and optimizing body weight to improve post‑FUAS fertility prospects, and recommends earlier referral to ART for patients with external adenomyosis. Thus, while older age may favor symptom durability, younger age appears advantageous for post‑treatment fertility, so “success” must be defined according to the patient’s primary goal. Observational data indicate that most FUAS candidates with adenomyosis are in their late 30 s to early 40 s (mean ages around 38–40 years)^31,32^. In a study of factors influencing treatment choice, age 31–40 years and a desire for fertility were among the key factors associated with choosing FUAS over hysterectomy^33^. MR‑guided FUS series for adenomyosis similarly enroll predominantly premenopausal women in their 30–40 s, with about one‑third remaining symptomatic at 6 months, prompting interest in models that incorporate age and imaging features to predict poor responders^34^. These patterns suggest that age not only affects biological response but also shapes patient selection, expectations (fertility vs. symptom control), and acceptable treatment aggressiveness. Higher estrogen milieu and more active myometrial invasion in younger women may promote progression or recurrence of adenomyosis after a partial ablation, reducing long‑term symptom control^31^. In older, perimenopausal women, lower hormonal stimulation and proximity to natural menopause may synergize with extensive ablation to yield more durable symptom improvement and lower re‑intervention rates^12,31^. Conversely, in younger women with fertility desire, operators may deliberately limit ablation volume to preserve myometrial integrity, trading off some long‑term symptom durability for reproductive safety, which can appear as an age effect in outcome analyses^12,31^.

The depth of adenomyosis within the myometrium strongly influences the technical feasibility, ablation efficiency, and clinical success of FUAS. MRI‑based classifications distinguish internal (subtype I, junctional zone/inner myometrium), external (subtype II, outer myometrium), intramural (subtype III), and full‑thickness (subtype IV) adenomyosis, which reflect the extent and depth of myometrial penetration^35^. Internal lesions tend to lie closer to the endometrium and often more centrally, whereas external and full‑thickness lesions extend toward the serosa, increasing sonication path length and proximity to bowel, sacrum, or abdominal wall^12,35^. For FUAS, “depth” effectively translates into sonication path length and relative position of the lesion between the endometrium and serosa. In a retrospective study of 238 internal and 167 external adenomyosis cases treated with FUAS, both groups showed significant improvement, but internal adenomyosis had a higher menorrhagia relief rate at 18 months (86.2% vs. 77.1%, *p* = 0.030), suggesting a more favorable clinical response in lesions closer to the junctional zone^36^. A 2024 series classifying patients into internal (I), external (II), intramural (III), and full‑thickness (IV) adenomyosis showed that full‑thickness lesions had significantly larger volumes and required longer irradiation time, longer total procedure time, and higher total energy input than internal or intramural lesions^35^. Full‑thickness disease also carried higher rates of post‑procedural abdominal pain and vaginal discharge from the treatment area than external disease, reflecting the greater tissue volume traversed and treated^35^. A 2024 narrative review of FUAS in adenomyosis highlights shorter sonication paths, internal or focal adenomyosis, low vascularity, fewer hyperintense foci on T2‑weighted MRI, and anterior location as key imaging predictors of favorable treatment outcomes^12^. Longer sonication paths (as in deep posterior or full‑thickness disease) increase energy loss and the risk of near‑field heating, limiting the safe acoustic power and thereby reducing achievable NPVR, which is a major determinant of long‑term symptom relief. Because internal/focal lesions usually have shorter paths and more compact geometry, they typically allow higher NPVR with less energy and shorter treatment times, translating into better volume reduction and more durable symptom control^12,37^. Clinically, this means that deep, posterior, and full‑thickness disease often receives a more conservative ablation than shallow, internal disease, which can lower success when success is defined as high NPVR and sustained symptom relief.

The distribution of adenomyosis within the uterus also influences the technical feasibility, ablation efficiency, and clinical success of FUAS. In the present study, uterine body adenomyosis showed lower FUAS efficacy than fundal disease, which is consistent with reports that lesion location and sonication path markedly influence energy transmission and achievable non‑perfused volume. Lesions situated deeper within the uterine body are often farther from the transducer and more likely to be traversed by abdominal fat, bowel, or scar tissue, all of which can attenuate or reflect ultrasound waves, reduce focal temperature, and limit effective ablation. Recent reviews and radiomics‑based analyses similarly identify greater lesion depth from the skin surface, posterior or deeply located adenomyosis, and more complex internal architecture as independent predictors of reduced NPV ratio and symptom response after FUAS^12,38,39^.

This study has several limitations that should be considered when interpreting the findings. First, it used a single-center, retrospective design, which may introduce information bias and limit the generalizability of the results to other institutions and patient populations. The overall sample size was relatively small (*n* = 250), and the distribution of cases across predictor categories was uneven, which may reduce statistical power and increase the risk of overfitting in the prediction model. In addition, there was no external validation cohort; thus, the nomogram has not yet been tested in independent populations, and its transportability to other clinical settings remains uncertain. Second, the endpoint used was primarily anatomical (ablation rate), which may not fully capture clinical success in terms of symptom relief, quality of life, or need for additional treatments. Patients selected for FUAS may also differ systematically from the broader population of individuals with adenomyosis (e.g., in terms of symptom severity, comorbidities, or treatment preferences), potentially introducing selection bias and further limiting the model’s applicability to all patients with adenomyosis. Third, although the follow-up period allowed assessment of short-term outcomes after FUAS, long-term follow-up was not systematically performed, and follow-up examinations were inconsistent among patients. As a result, we were unable to evaluate the durability of treatment response, long-term recurrence, or downstream clinical outcomes, and the model reflects only short-term anatomical efficacy rather than long-term clinical benefit. Finally, as with all retrospective observational studies, there is a risk of residual and unmeasured confounding; some relevant clinical, imaging, or procedural variables may not have been recorded or may have been incompletely captured in the medical records, which could affect both model development and the observed associations. Prospective, multicenter studies with larger, more diverse cohorts, standardized follow-up, clinically meaningful endpoints, and external validation are needed to confirm and refine this prediction model and to support its broader clinical application.

In conclusion, conventional clinical parameters, anatomical and morphological indicators, and FUAS treatment parameters have the potential to predict FUAS outcomes in patients with adenomyosis. Age, depth of adenomyosis, and uterine body lesions were significant predictors of FUAS efficacy in patients with adenomyosis. The nomogram can accurately predict the success rate of adenomyosis after undergoing FUAS. It provides an early, accurate, non-invasive, and comprehensive evaluation tool for assessing FUAS sensitivity in patients with adenomyosis and offers an objective basis for individualized treatment.

## Supplementary Information

Below is the link to the electronic supplementary material.

Supplementary Material 1

## Data Availability

All data generated or analyzed during this study are included in this published article.
