# Supplementary material for: Development and validation of a clinical prediction model for the success of focused ultrasound ablation system for the treatment of adenomyosis
Source: Sci Rep. 2026 Jun 2;16:25054. doi: 10.1038/s41598-026-48587-z (PMC13462748; doi:10.1038/s41598-026-48587-z)
Supplement: Supplementary file 1 [file 41598_2026_48587_MOESM1_ESM.docx]

**Supplementary Table S1.** Multicollinearity analysis

| Variable | GVIF | Df | GVIF_1_2Df |
| --- | --- | --- | --- |
| AGE | 1.794 | 1 | 1.339 |
| Depth of adenomyosis lesions | 1.206 | 1 | 1.098 |
| Maximum power | 1.773 | 1 | 1.331 |
| Average power | 2 | 1 | 1.414 |
| Number of births | 2.367 | 4 | 1.114 |
| Anemia | 1.188 | 1 | 1.09 |
| Fertility requirements | 2.31 | 1 | 1.52 |
| Uterine position | 1.422 | 2 | 1.092 |
| Lesion position | 1.223 | 2 | 1.052 |
| Dysmenorrhea score | 1.193 | 1 | 1.092 |
| Type | 1.692 | 5 | 1.054 |

**Supplementary Table S2.** Model coefficients table

| Variable | Estimate | Std_Error | z_value | Pr_z |
| --- | --- | --- | --- | --- |
| (Intercept) | -6.7607857 | 6.3124118 | -1.071 | 0.2842 |
| AGE | 0.0953245 | 0.0386661 | 2.465 | 0.0137 |
| Depth of adenomyosis lesions | 0.0294316 | 0.0140577 | 2.094 | 0.0363 |
| Maximum power 400 | 2.1921127 | 1.360923 | 1.611 | 0.1072 |
| Average power | 0.0006726 | 0.0169089 | 0.04 | 0.9683 |
| Number of births 1 | 1.3514929 | 0.7057818 | 1.915 | 0.0555 |
| Number of births 2 | 1.3762593 | 0.8109151 | 1.697 | 0.0897 |
| Number of births 3 | 0.9065261 | 1.3474995 | 0.673 | 0.5011 |
| Number of births 4 | -15.7342074 | 1455.397847 | -0.011 | 0.9914 |
| Anemia 1 | 0.4606374 | 0.3651336 | 1.262 | 0.2071 |
| Fertility requirements 1 | 1.1315825 | 0.5978044 | 1.893 | 0.0584 |
| Uterine position 1 | -0.1839569 | 0.4381309 | -0.42 | 0.6746 |
| Uterine position 2 | -15.1212549 | 1455.397749 | -0.01 | 0.9917 |
| Uterine body | -1.3942753 | 0.6310135 | -2.21 | 0.0271 |
| Diffuse lesions | -1.8350432 | 1.5840009 | -1.158 | 0.2467 |
| Dysmenorrhea score | -0.0518813 | 0.0706663 | -0.734 | 0.4628 |
| Type 2 | -0.2186381 | 0.5786305 | -0.378 | 0.7055 |
| Type 3 | -0.1377906 | 0.9570146 | -0.144 | 0.8855 |
| Type 4 | -0.5864657 | 0.4370067 | -1.342 | 0.1796 |
| Type 5 | -1.8135263 | 1.3197965 | -1.374 | 0.1694 |
| Type 6 | -1.2041513 | 0.6650622 | -1.811 | 0.0702 |

**Supplementary Figure S1.** Bootstrap calibration curve in validation set.
